# What students pursue matters: longitudinal associations between goal contents and life skills in university physical education

**DOI:** 10.3389/fpsyg.2026.1964768

**Published:** 2026-09-25

**Authors:** WooYoung Joo, Yeonhak Jung, Youmin Son

**Affiliations:** 1 Institute of Sports Science, Pukyong National University, Busan, Republic of Korea; 2 Department of Kinesiology, California State University, Northridge, Los Angeles, CA, United States; 3 Department of Physical Education, Korea University, Seoul, Republic of Korea

**Keywords:** emerging adulthood, extrinsic goal, intrinsic goal, life skills, longitudinal design

## Abstract

**Introduction:**

Research on goal contents has largely focused on why students engage in physical activity, with less attention to what they pursue through it. This study examined the prospective and possible reverse-direction associations between intrinsic and extrinsic goal contents and five life skills among students in university physical education.

**Methods:**

A two-wave cross-lagged panel design was employed over one academic semester with 347 students nested within 26 university physical education class sections. Participants completed self-report measures of intrinsic and extrinsic goals and five domains of life skills (teamwork, goal setting, time management, social skills, and leadership) at the beginning (T1) and end (T2) of the semester.

**Results:**

After accounting for prior levels of the study variables and sex, T1 intrinsic goals showed prospective associations with T2 time management and leadership in the unadjusted results, but only the association with time management remained statistically significant after the Holm correction. T1 extrinsic goals were not significantly associated with any of the five life skills at T2. Regarding the reverse-direction associations, T1 leadership was negatively associated with T2 extrinsic goals in the unadjusted results, although this association did not remain statistically significant after the Holm correction. None of the remaining reverse-direction paths were statistically significant.

**Discussion:**

These findings suggest that intrinsic goals may be prospectively associated with time management and, more tentatively, leadership, rather than uniformly with all five life skills. They also provide initial insight into the prospective direction of the associations between goal contents and life skills in university physical education.

## 1 Introduction

Emerging adulthood represents a critical developmental period characterized by increased autonomy, identity exploration, and the transition toward independent life roles (Arnett, 2000). During this stage, university students, as a major subgroup of emerging adults, face multiple developmental tasks, including career preparation, psychological independence, and the formation of interpersonal relationships. As external structures and supervision decline, individuals are increasingly required to regulate their own behaviors and navigate complex social environments (Schulenberg and Schoon, 2012). Consequently, higher education is expected not only to develop students’ academic and vocational knowledge but also to provide educational experiences that foster the self-regulatory and socioemotional capacities required for adult life.

One construct that captures these adaptive capacities is life skills. Life skills refer to the psychological and social competencies that enable individuals to function effectively across academic, occupational, and interpersonal domains (Danish et al., 1993). These competencies include goal setting, social skills, time management, teamwork, and leadership (Cronin and Allen, 2017). Such competencies may play a crucial role in helping university students manage academic demands, interact effectively with others, and adapt to the various psychological and social challenges they encounter during university life. Research involving university students has shown that a range of life skills are positively associated with psychological wellbeing and academic self-efficacy, while goal setting, time management, and leadership are also positively associated with students’ predicted academic performance (Cronin et al., 2021). In addition, a preliminary intervention study found that university students who participated in life skills training showed improvements in mental health indicators (Savoji and Ganji, 2013). Together, these findings suggest that life skills represent important educational competencies that may support not only university students’ academic functioning but also their psychological health and adjustment to university life.

University physical education provides a valuable context in which students can experience and develop life skills because it combines structured physical activity with educational experiences (Zadarko-Domaradzka et al., 2024). Engagement in physical activity inherently involves goal pursuit, persistence, emotional regulation, and repeated adaptation to success and failure (Gould and Carson, 2008). Team-based activities further require cooperation, communication, and shared responsibility, whereas individual activities demand self-regulation and sustained effort. University physical education may also afford students some degree of autonomy over their activity choices and modes of participation, allowing them to assign personal meaning to their participation based on their own goals and values (Teixeira et al., 2012). A recent study of students enrolled in a university physical education course found increases in personal, interpersonal, and overall life skills following course participation (Kaneda et al., 2026). Together, this conceptual and emerging empirical evidence highlights university physical education as a meaningful educational setting for supporting students’ life skills (Chen, 2023). However, existing research on life skills has focused primarily on youth sport and physical education in secondary schools (Cronin et al., 2020; Vahdani et al., 2025), whereas empirical research examining university students’ life skills within university physical education remains relatively limited (Kaneda et al., 2026). Accordingly, greater research attention should be devoted to life skills within university physical education.

Research grounded in Self-Determination Theory (SDT; Ryan and Deci, 2017) has provided substantial insights into the mechanisms underlying adaptive outcomes in physical activity contexts. Within SDT, the “why” and the “what” of behavior provide distinct but complementary perspectives for understanding individuals’ adaptive functioning. The “why” of behavior refers to individuals’ reasons for engaging in an activity and the corresponding quality of behavioral regulation, commonly examined along a continuum ranging from controlled regulation to autonomous regulation (Ryan and Deci, 2017). Alongside behavioral regulation, basic psychological need is understood as a related psychological process that helps explain the relationships among social contexts, motivation, and adaptive functioning. In contrast, the “what” of behavior refers to the contents of the goals that individuals pursue through an activity. Both why individuals engage in an activity and what they pursue through that activity are relevant to their adaptive functioning.

However, research on physical activity and life skills has primarily focused on factors related to the “why” of behavior, including intrinsic motivation and psychological need satisfaction. Specifically, these motivational factors have been associated with adaptive outcomes, including life skills and wellbeing (Cronin et al., 2019; Freire et al., 2023; Ortiz-Rodríguez et al., 2024; Zheng et al., 2023). By contrast, relatively limited attention has been directed toward what individuals pursue through their participation, namely, how goal contents are associated with life skills. Therefore, to complement the existing emphasis on behavioral regulation and need satisfaction, greater attention should be directed toward the contents of the goals that students pursue in university physical education.

Goal Contents Theory (GCT; Deci and Ryan, 2000; Kasser and Ryan, 1996), a sub-theory within SDT, provides a useful theoretical framework for understanding what university students pursue throughout their participation in university physical education, namely, their goal contents. GCT distinguishes between intrinsic and extrinsic goal contents. In physical activity contexts, intrinsic goals are directed toward self-development, health, and meaningful relationships and, in the present study, comprise skill development (“to become good at an activity”), health management (“to increase my energy level”), and social affiliation (“to have meaningful interactions with my classmates”). By contrast, extrinsic goals focus on external evaluation and approval and, in the present study, comprise social recognition (“to gain recognition from my classmates”) and body image (“to improve the attractiveness of my appearance and body shape”; Sebire et al., 2008, 2009). These intrinsic and extrinsic goals may be associated with different psychological and behavioral outcomes (Vansteenkiste et al., 2010).

Indeed, the pursuit of intrinsic goals has been positively associated with psychological need satisfaction and autonomous forms of motivation (Sibley and Bergman, 2018), life satisfaction (Martela et al., 2019), vitality (Unanue et al., 2014), mindfulness (Brown and Kasser, 2005), and pro-environment and prosocial behaviors (Fu et al., 2018; Unanue et al., 2016). Conversely, the pursuit of extrinsic goals has been associated with psychological need frustration (Bradshaw et al., 2021; Nishimura et al., 2021), controlled motivation (Jeno et al., 2018), and various indicators of psychological maladjustment (Kasser et al., 2014; Ryan et al., 1999).

The adaptive role of intrinsic goals has also received empirical support in physical activity contexts. Intrinsic goals have been associated with greater wellbeing, sustained engagement, psychological need satisfaction, and autonomous motivation (Codina et al., 2024; Sebire et al., 2011). Longitudinal research has further shown that increases in intrinsic goals relative to extrinsic goals are accompanied by corresponding increases in autonomous motivation over time (Behzadnia et al., 2022; Gunnell et al., 2014). Beyond these general psychological and motivational outcomes, intrinsic and extrinsic goals may also differ in their associations with life skills because of their distinct orientations. For example, students who pursue skill development and health management may be more likely to set goals for improvement, monitor their performance, and continuously manage their participation and effort. These processes are conceptually aligned with goal setting and time management, respectively. Students who pursue social affiliation may be more likely to communicate and cooperate with their classmates and seek to understand others’ perspectives; thus, this goal may be closely connected to teamwork and social skills. Moreover, the initiative, responsibility, coordination of group members, and support for others involved in pursuing both self-improvement and meaningful relationships are conceptually aligned with leadership. Thus, the processes involved in pursuing intrinsic goals may be more directly aligned with students’ perceived development across all five life skills.

By contrast, extrinsic goals focused on social recognition and body image direct attention toward outcomes defined by others’ evaluations or social comparison. Although these goals may also motivate students’ participation and effort, they may place relatively less direct emphasis on the self-regulatory and interpersonal processes represented by goal setting, time management, teamwork, social skills, and leadership. Therefore, rather than assuming that extrinsic goals are necessarily negatively associated with life skills, they may be expected to demonstrate weaker or less consistent associations than intrinsic goals.

Existing GCT research in physical activity has primarily investigated behavioral regulation, psychological need satisfaction, wellbeing, and physical activity participation, whereas little is known about how goal contents are associated with broader educational outcomes such as life skills. More specifically, research directly examining the relationship between goal contents and life skills in physical activity contexts is lacking, particularly among emerging adults participating in university physical education. Therefore, it remains to be determined whether the adaptive associations of intrinsic goals extend beyond the motivational and psychological outcomes examined in previous research to life skills and how extrinsic goals are associated with these skills.

Furthermore, most previous studies examining life skills development have relied on cross-sectional designs (Cronin et al., 2018, 2021), which are limited in their ability to establish temporal ordering among variables or examine longitudinal associations while accounting for prior levels of life skills. Although some longitudinal research on goal contents has been conducted, these studies have primarily focused on motivational outcomes such as autonomous motivation (Behzadnia et al., 2022; Gunnell et al., 2014), and the longitudinal associations between goal contents and life skills have not been directly examined. This limitation is particularly important because life skills are developmental competencies that may develop gradually through repeated participation experiences rather than emerge as immediate outcomes (Cronin et al., 2020; Lim et al., 2026). Consequently, it remains unclear whether intrinsic and extrinsic goals are associated with subsequent life skills after accounting for initial levels of life skills. Addressing this temporal relationship requires longitudinal research that investigates the prospective associations between goal contents and life skills while accounting for their initial levels.

The association between goal contents and life skills may also be bidirectional rather than strictly unidirectional. A systematic review of sport-based life skills programs showed that programs in which life skills development and transfer were evidenced included self-regulatory and interpersonal competencies such as goal setting, planning, self-management, health skills, teamwork, communication, social support, and leadership (Williams et al., 2022). By applying these skills, students may come to value the personal growth, wellbeing, and social connection afforded by their participation, which may, in turn, be associated with a stronger subsequent orientation over time toward intrinsic goals such as skill development, health management, and social affiliation (Vansteenkiste et al., 2010). Thus, it is necessary to consider not only whether goal contents may be associated with subsequent life skills but also whether life skills may be associated with later goal contents. To our knowledge, no previous study has examined prospective pathways from life skills to goal contents in physical activity contexts, nor has any study simultaneously investigated the forward and reverse associations between these variables. Taken together, these research gaps leave the temporal direction of the associations between goal contents and life skills unclear.

Accordingly, the present study examined the longitudinal associations between intrinsic and extrinsic goal contents and life skills among university students participating in university physical education. Specifically, using a two-wave cross-lagged panel design, we examined the forward associations from T1 intrinsic and extrinsic goals to the five life skills at T2 and the reverse associations from each life skill at T1 to intrinsic and extrinsic goals at T2, while accounting for the prior levels of all constructs. Whereas prior SDT-based research has primarily focused on behavioral regulation, which represents the “why” of participation, and related basic psychological need processes, goal contents, which represent the “what” individuals pursue through participation, have received comparatively limited attention as factors associated with life skills. Thus, the present study sought to address this gap by examining both forward and reverse prospective associations between goal contents and life skills.

Based on GCT and the theoretical rationale presented above, T1 intrinsic goals were expected to be positively associated with each of the five life skills at T2. In contrast, because extrinsic goals place relatively less direct emphasis on the self-regulatory and interpersonal processes associated with life skills, T1 extrinsic goals were expected to show limited prospective associations with life skills at T2. Given the lack of direct longitudinal evidence regarding the associations between goal contents and life skills, particularly the absence of previous research examining pathways from T1 life skills to T2 goal contents, we explored whether each life skill at T1 was associated with intrinsic and extrinsic goals at T2 without specifying the expected directions of these associations in advance. The model examined in the present study is presented in Figure 1.

**Figure 1:**
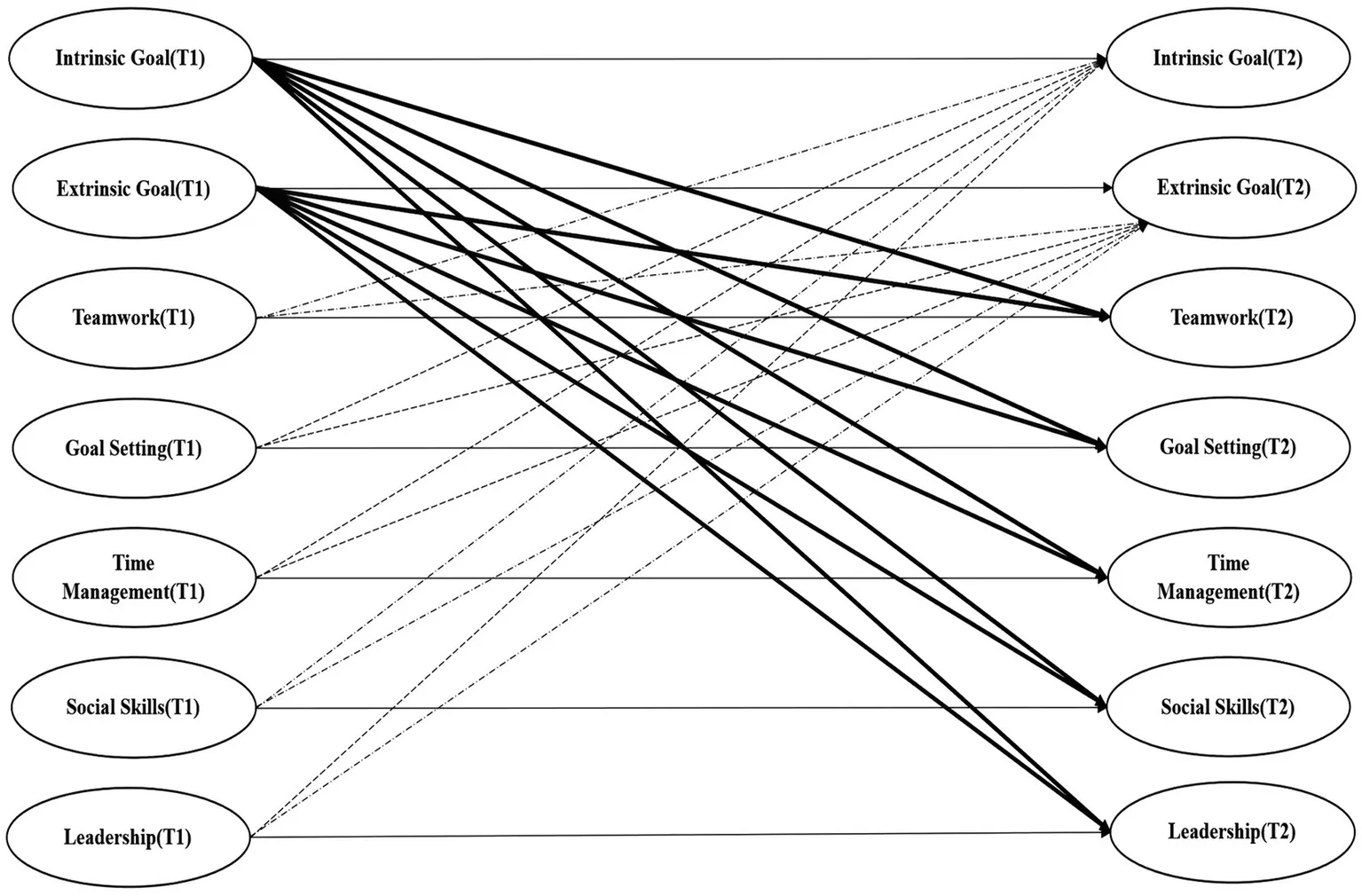
Two-wave cross-lagged panel model examining the prospective and reverse-direction associations between intrinsic and extrinsic goals and life skills. Life skills included teamwork, goal setting, time management, social skills, and leadership. Bold solid lines represent the forward paths from T1 goal contents to T2 life skills, thin solid lines represent autoregressive paths, and dashed lines represent the exploratory reverse-direction paths from T1 life skills to T2 goal contents.

## 2 Materials and methods

### 2.1 Participants

Participants were 393 undergraduate students enrolled in 26 general physical education class sections representing 12 activity types in South Korea at T1. The 26 class sections were taught by 14 instructors, with some instructors teaching more than one class section during the semester. Of these, 347 students (*M_age_* = 22.02 years, *SD* = 2.08; 203 males and 144 females) provided matched responses across both measurement waves and were retained for the final longitudinal analyses, yielding a retention rate of 88.3%. Participants represented diverse academic majors, including humanities and social sciences (*n* = 129), natural sciences (*n* = 42), engineering (*n* = 85), arts and sports sciences (*n* = 73), and other fields (*n* = 18). Students participated in 12 types of physical activity courses, including yoga, swimming, badminton, fitness training, athletics, soccer, softball, basketball, table tennis, squash, camping, and skating.

### 2.2 Procedure

Data were collected at two time points over one academic semester. At the beginning of the semester (T1; weeks 1–2), students completed questionnaires assessing goal contents and perceived life skills during regularly scheduled physical education classes. The same questionnaires were administered at the end of the semester (T2; weeks 15–16). The approximately 14-week interval corresponded to a naturally occurring academic semester and was selected to examine prospective associations across a sustained period of participation in university physical education. A school-term interval has also been used to examine changes in life skills and their motivational correlates in physical education (Cronin et al., 2020).

Attrition between T1 and T2 was primarily attributable to course withdrawal, absence on the day of data collection, or incomplete survey responses, or responses that could not be matched across waves. Only participants who provided complete and matched responses at both time points were included in the longitudinal analyses. Participation was voluntary, and informed consent was obtained from all participants in accordance with institutional ethical guidelines. Responses across the two waves were matched using student identification numbers, which were removed from the analytic dataset after the matching process was completed. Each questionnaire required approximately 10–15 min to complete.

The study was reviewed and approved by the Institutional Review Board of Korea University (Approval No. KUIRB-2025-0355-01).

### 2.3 Measures

All questionnaires were administered in Korean. The Goal Content for Exercise Questionnaire was translated using a forward- and back-translation procedure. First, the original English items were translated into Korean by a member of the research team. An independent bilingual expert who had not participated in the initial translation then back-translated the Korean items into English. The original and back-translated versions were compared, and discrepancies in wording or meaning were discussed and revised. Subsequently, a panel comprising two professors of sport psychology and one researcher with a doctoral degree in sport psychology reviewed the translated items for linguistic clarity, conceptual equivalence, and suitability for Korean university physical education contexts. The finalized Korean version was used in the present study. The Korean version of the Life Skills Scale for Sport had been previously translated and validated by Lim et al. (2019).

#### 2.3.1 Goal contents

Goal contents were assessed using the 20-item Goal Content for Exercise Questionnaire (Sebire et al., 2008), which was developed within GCT (Kasser and Ryan, 1996). The questionnaire comprises five four-item subscales: social affiliation (e.g., “to interact meaningfully with classmates”), health management (e.g., “to improve my overall health”), skill development (e.g., “to acquire new skills related to the activity”), social recognition (e.g., “to gain social respect from classmates”), and body image (e.g., “to improve my physical appearance”). Social affiliation, health management, and skill development represent intrinsic goal contents, whereas social recognition and body image represent extrinsic goal contents. Participants responded using a 7-point scale from 1 (strongly disagree) to 7 (strongly agree).

All 20 original GCEQ items and all five goal-content dimensions were retained. Item scores were averaged within each subscale. Consistent with previous higher-order specifications of the GCEQ (Behzadnia et al., 2022; Sebire et al., 2008), social affiliation, health management, and skill development subscale scores served as indicators of the higher-order intrinsic-goal factor, whereas social recognition and body image subscale scores served as indicators of the higher-order extrinsic-goal factor.

The confirmatory factor analysis indicated an overall acceptable fit, *χ*^2^(160) = 553.335, CFI = 0.921, TLI = 0.906, RMSEA = 0.082, 90% CI [0.074, 0.090], and SRMR = 0.051. All standardized factor loadings were statistically significant (*p* < 0.001), ranging from 0.878 to 0.981 for social affiliation, 0.589 to 0.946 for health management, 0.781 to 0.923 for skill development, 0.779 to 0.941 for social recognition, and 0.864 to 0.970 for body image. Cronbach’s α values for intrinsic goals were 0.89 at T1 and 0.93 at T2, whereas those for extrinsic goals were 0.92 at T1 and 0.92 at T2.

#### 2.3.2 Life skills

Life skills were assessed using the Korean version of the Life Skills Scale for Sport (KLSSS; Lim et al., 2019), adapted from the original Life Skills Scale for Sport developed by Cronin and Allen (2017). The original LSSS consists of 43 items assessing eight life-skill domains, whereas the validated Korean version comprises 18 items across five subdomains: teamwork (3 items), goal setting (4 items), time management (3 items), social skills (4 items), and leadership (4 items). Sample items include “I work with others for the benefit of the team” (teamwork), “I set challenging goals” (goal setting), “I manage my time effectively” (time management), “I communicate effectively with others” (social skills), and “I know how to motivate others” (leadership). Participants responded on a 5-point Likert scale ranging from 1 (strongly disagree) to 5 (strongly agree).

The confirmatory factor analysis indicated good model fit, *χ*^2^(125) = 230.771, CFI = 0.961, TLI = 0.953, RMSEA = 0.049, 90% CI [0.039, 0.059], and SRMR = 0.045. All standardized factor loadings were statistically significant (*p* < 0.001), ranging from 0.710 to 0.813 for teamwork, 0.750 to 0.812 for goal setting, 0.766 to 0.917 for time management, 0.664 to 0.882 for social skills, and 0.774 to 0.865 for leadership. To examine the empirical distinctiveness of the five life-skills domains, the hypothesized correlated five-factor model reported above was compared with an alternative one-factor model in which all 18 items loaded on a single life-skills factor. The one-factor model showed poor fit, *χ*^2^(135) = 792.292, CFI = 0.759, TLI = 0.727, RMSEA = 0.118, 90% CI [0.111, 0.127], and SRMR = 0.077. Thus, the substantially better fit of the five-factor model supported the empirical distinction among teamwork, goal setting, time management, social skills, and leadership despite their substantial intercorrelations. Internal consistency estimates in the present study were acceptable to excellent. Cronbach’s α coefficients at T1 were as follows: teamwork (*α* = 0.78), goal setting (*α* = 0.87), time management (*α* = 0.88), social skills (*α* = 0.87), and leadership (*α* = 0.89). Corresponding coefficients at T2 were 0.82, 0.90, 0.89, 0.91, and 0.92, respectively.

### 2.4 Data analyses

Data analyses were conducted using SPSS version 21.0 and Mplus version 8.11. Preliminary analyses, including descriptive statistics, reliability estimates (Cronbach’s α), and bivariate correlations, were performed in SPSS. All longitudinal structural analyses were conducted in Mplus. The longitudinal analyses were based on the 347 participants who provided complete and matched responses at both waves. Because the analytic sample contained only complete and matched responses, FIML was not used to handle missing data.

To evaluate potential dependency arising from students being nested within class sections, unconditional random-intercept models were estimated for the principal study variables. Intraclass correlation coefficients (ICCs) were calculated as the proportion of total variance attributable to between-class differences (Hedges and Hedberg, 2007). Design effects were subsequently calculated as 1 + (m̄ − 1) × ICC (Kish, 1965), where m̄ represents the average class size. Given that 347 students were nested within 26 class sections, the average class size was 13.35.

Model fit was evaluated using the comparative fit index (CFI), Tucker–Lewis index (TLI), root mean square error of approximation (RMSEA), and standardized root mean square residual (SRMR). Values of 0.90 or higher for CFI and TLI were considered indicative of acceptable fit, with values of 0.95 or higher reflecting good fit. RMSEA and SRMR values of 0.08 or lower indicated acceptable fit, with values of 0.06 or lower reflecting good fit (Hu and Bentler, 1999).

Prior to testing the structural model, longitudinal measurement invariance of intrinsic goals, extrinsic goals, and the five life-skills factors was examined simultaneously across the two waves. Intrinsic goals were represented by the social affiliation, health management, and skill development subscale scores, whereas extrinsic goals were represented by social recognition and body image subscale scores. A sequence of increasingly constrained models—configural, metric, and scalar invariance—was estimated to ensure that the constructs were measured equivalently over time. All latent factors were allowed to correlate within each wave. Residuals of corresponding indicators were permitted to correlate across waves to account for persistent indicator-specific variance. Following Cheung and Rensvold (2002) and Chen (2007), invariance was supported when changes in CFI (ΔCFI) were less than or equal to 0.010 and changes in RMSEA (ΔRMSEA) were less than or equal to 0.015. Changes in SRMR (ΔSRMR) of 0.030 or less for metric invariance and 0.010 or less for scalar invariance were considered acceptable. To examine shared variance and discriminant validity among the five life-skills domains, separate correlated five-factor measurement models were estimated at T1 and T2. Standardized latent-factor correlations were obtained from these CFA models. In addition, heterotrait-monotrait ratios of correlations (HTMT) were calculated from the item-level correlation matrices as the mean of the absolute correlations between indicators of different constructs divided by the geometric mean of the average correlations among indicators within each construct. HTMT values below 0.85 and 0.90 were considered indicative of discriminant validity according to conservative and more liberal criteria, respectively (Henseler et al., 2015).

Prospective and reverse-direction associations between goal contents and life skills were examined using a two-wave cross-lagged panel model (CLPM) estimated with maximum likelihood estimation with robust standard errors (MLR), which accounts for potential non-normality in the data. The model included autoregressive paths to account for temporal stability and cross-lagged paths from T1 intrinsic and extrinsic goals to each of the five life-skills factors at T2. Reverse paths from T1 life skills to T2 goal contents were also specified to explore possible reverse-direction associations. Within-wave covariances among the T1 factors and residual covariances among the T2 factors were freely estimated. Sex was retained as the only covariate because of its theoretical relevance to potential differences in goal contents and life skills. It was specified as a predictor of all T2 variables, including intrinsic and extrinsic goals and the five life-skills factors. To address multiplicity across the cross-lagged path tests in the primary latent CLPM, Holm’s sequentially rejective procedure (Holm, 1979) was used to control the familywise error rate. Corrections were applied separately to three theoretically defined inferential families: (a) the five prospective paths from T1 intrinsic goals to T2 life skills, (b) the five prospective paths from T1 extrinsic goals to T2 life skills, and (c) the ten exploratory reverse-direction paths from T1 life skills to T2 intrinsic and extrinsic goals. Both unadjusted and Holm-adjusted *p* values for the primary latent CLPM are reported in Supplementary Table S2.

Because the number of class sections was limited relative to the complexity of the full latent CLPM, seven parsimonious cluster-robust sensitivity analyses were conducted in Mplus using observed composite scores. Five life-skill–specific models examined prospective associations from goal contents to life skills. In each model, one T2 life-skill outcome was regressed on its corresponding T1 score, T1 intrinsic and extrinsic goals, and sex. Two additional goal-content–specific models examined the reverse-direction associations. T2 intrinsic goals and T2 extrinsic goals were each regressed on their corresponding T1 goal content, the five T1 life skills, and sex. Dependency among students within the 26 class sections was accounted for using TYPE = COMPLEX with the MLR estimator, which provides sandwich standard errors that are robust to the nonindependence of observations within class sections.

## 3 Results

### 3.1 Descriptive and correlation analyses

Table 1 presents the descriptive statistics and bivariate correlations for the study variables. Intrinsic goal content showed relatively high mean scores at both T1 (*M* = 6.09, *SD* = 0.74) and T2 (*M* = 6.24, *SD* = 0.72), whereas extrinsic goal showed moderate mean scores at T1 (*M* = 4.64, *SD* = 1.39), and T2 (*M* = 4.83, *SD* = 1.41). Mean scores for the five life skills ranged from 3.91 to 4.33 at T1 and from 4.17 to 4.47 at T2, indicating moderate-to-high levels across both measurement occasions. Skewness values ranged from −0.99 to 0.04, and kurtosis values from −1.06 to 2.05, indicating no substantial departures from univariate normality. All correlations were positive and statistically significant, ranging from 0.13 to 0.81; the weakest associations were significant at *p* < 0.05, whereas most correlations were significant at *p* < 0.01.

**Table 1:** Descriptive statistics and correlations among model variables.

Variable	M ± SD	1	2	3	4	5	6	7	8	9	10	11	12	13	14
1. T1_IG	6.09 ± 0.74	–													
2. T1_EG	4.64 ± 1.39	0.47	–												
3. T1_TW	4.33 ± 0.58	0.49	0.18	–											
4. T1_GS	4.15 ± 0.69	0.50	0.22	0.59	–										
5. T1_TM	3.92 ± 0.84	0.49	0.26	0.49	0.67	–									
6. T1_SS	4.16 ± 0.65	0.56	0.26	0.58	0.63	0.52	–								
7. T1_LS	3.91 ± 0.74	0.57	0.33	0.57	0.61	0.59	0.74	–							
8. T2_IG	6.24 ± 0.72	0.54	0.28	0.39	0.36	0.33	0.42	0.36	–						
9. T2_EG	4.83 ± 1.41	0.26	0.64	0.15	0.16	0.13^*^	0.16	0.18	0.39	–					
10. T2_TW	4.47 ± 0.60	0.38	0.16	0.46	0.37	0.30	0.41	0.33	0.61	0.17	–				
11. T2_GS	4.30 ± 0.69	0.37	0.18	0.41	0.50	0.46	0.46	0.43	0.60	0.19	0.67	–			
12. T2_TM	4.18 ± 0.81	0.40	0.23	0.36	0.43	0.49	0.43	0.41	0.50	0.23	0.53	0.70	–		
13. T2_SS	4.32 ± 0.69	0.36	0.20	0.42	0.37	0.32	0.47	0.41	0.64	0.25	0.71	0.70	0.61	–	
14. T2_LS	4.17 ± 0.76	0.41	0.25	0.41	0.43	0.40	0.51	0.50	0.62	0.30	0.61	0.71	0.69	0.81	–

IG, intrinsic goal; EG, extrinsic goal; TW, teamwork; GS, goal setting; TM, time management; SS, social skills; LS, leadership; T1, Time 1; T2, Time 2; M, mean; SD, standard deviation. Goal content variables were measured on a 7-point scale; life skill variables were measured on a 5-point scale. All correlations were significant at *p* < 0.05 or *p* < 0.01.

### 3.2 Classroom clustering

The ICCs and corresponding design effects for the principal study variables are presented in Supplementary Table S1. Across the two waves, ICCs ranged from 0.000 to 0.116, indicating that between 0 and 11.6% of the variance in the study variables was attributable to differences between class sections. The corresponding design effects ranged from 1.00 to 2.43. Classroom clustering was most pronounced for T1 time management (ICC = 0.116, design effect = 2.43) and T1 leadership (ICC = 0.092, design effect = 2.13), whereas clustering was negligible for several other variables.

### 3.3 Model fit and measurement invariance testing

Longitudinal measurement invariance was examined sequentially across the two measurement occasions. As shown in Table 2, the configural model demonstrated acceptable fit, *χ*^2^(877) = 1607.746, CFI = 0.920, TLI = 0.905, RMSEA = 0.049, 90% CI [0.045, 0.053], and SRMR = 0.060. Constraining the factor loadings to equality across time did not meaningfully reduce model fit, *χ*^2^(893) = 1626.376, CFI = 0.919, TLI = 0.907, RMSEA = 0.049, 90% CI [0.045, 0.052], and SRMR = 0.061 (ΔCFI = −0.001, ΔRMSEA = 0.000, ΔSRMR = 0.001). The scalar invariance model also demonstrated comparable fit, *χ*^2^(909) = 1653.465, CFI = 0.918, TLI = 0.907, RMSEA = 0.049, 90% CI [0.045, 0.052], and SRMR = 0.062 (ΔCFI = −0.001, ΔRMSEA = 0.000, ΔSRMR = 0.001). Changes in CFI were below the recommended threshold of 0.01, and changes in RMSEA and SRMR were also negligible, supporting full scalar invariance across T1 and T2 (Cheung and Rensvold, 2002).

**Table 2:** Measurement invariance testing.

CFA Model	*χ^2^* (df)	RMSEA	SRMR	CFI	TLI	ΔCFI	ΔRMSEA	ΔSRMR
Configural invariance	1607.746 (877)	0.049	0.060	0.920	0.905	–	–	–
Metric invariance	1626.376 (893)	0.049	0.061	0.919	0.907	−0.001	0.000	0.001
Scalar invariance	1653.465 (909)	0.049	0.062	0.918	0.907	−0.001	0.000	0.001

ΔCFI ≤ 0.01 indicates invariance (Cheung and Rensvold, 2002).

The standardized latent-factor correlations and HTMT values for the five life-skills domains are presented in Supplementary Table S3. At T1, the latent-factor correlations ranged from 0.554 to 0.813, whereas the HTMT values ranged from 0.586 to 0.840. At T2, the latent-factor correlations ranged from 0.587 to 0.852, whereas the HTMT values ranged from 0.618 to 0.883. The strongest association at both measurement occasions was observed between social skills and leadership (latent correlations = 0.813 and 0.852; HTMT = 0.840 and 0.883 at T1 and T2, respectively). All HTMT values were below the more liberal 0.90 criterion, although the HTMT value between social skills and leadership at T2 exceeded the conservative 0.85 criterion. These findings generally supported the empirical distinction among the five life-skills domains while indicating substantial shared variance, particularly between social skills and leadership (see Table 3).

**Table 3:** Cross-lagged model results.

Paths	*β*	SE	*z*	*p*
Autoregressive paths				
**T1 intrinsic goals → T2 intrinsic goals**	**0.616**	**0.280**	**2.20**	**0.028**
**T1 extrinsic goals → T2 extrinsic goals**	**0.765**	**0.093**	**8.27**	***p* < 0.001**
T1 teamwork → T2 teamwork	0.208	0.189	1.10	0.271
**T1 goal setting → T2 goal setting**	**0.354**	**0.112**	**3.18**	**0.001**
**T1 time management → T2 time management**	**0.288**	**0.070**	**4.10**	***p* < 0.001**
T1 social skills → T2 social skills	0.120	0.224	0.53	0.593
**T1 leadership → T2 leadership**	**0.244**	**0.103**	**2.36**	**0.018**
Cross-lagged paths				
T1 intrinsic goals → T2 teamwork	0.519	0.311	1.67	0.095
T1 intrinsic goals → T2 goal setting	0.320	0.197	1.63	0.103
**T1 intrinsic goals → T2 time management**	**0.399**	**0.101**	**3.96**	***p* < 0.001**
T1 intrinsic goals → T2 social skills	0.579	0.429	1.35	0.177
**T1 intrinsic goals → T2 leadership**	**0.463**	**0.208**	**2.22**	**0.026**
T1 extrinsic goals → T2 teamwork	−0.202	0.241	−0.84	0.403
T1 extrinsic goals → T2 goal setting	−0.084	0.171	−0.49	0.624
T1 extrinsic goals → T2 time management	−0.068	0.116	−0.59	0.559
T1 extrinsic goals → T2 social skills	−0.209	0.355	−0.59	0.556
T1 extrinsic goals → T2 leadership	−0.102	0.206	−0.50	0.619
Reverse-direction paths				
T1 teamwork → T2 intrinsic goals	0.068	0.173	0.39	0.695
T1 goal setting → T2 intrinsic goals	0.038	0.105	0.37	0.713
T1 time management → T2 intrinsic goals	−0.060	0.097	−0.62	0.536
T1 social skills → T2 intrinsic goals	0.155	0.131	1.19	0.235
T1 leadership → T2 intrinsic goals	−0.214	0.115	−1.86	0.064
T1 teamwork → T2 extrinsic goals	0.099	0.096	1.03	0.301
T1 goal setting → T2 extrinsic goals	0.144	0.119	1.21	0.225
T1 time management → T2 extrinsic goals	−0.161	0.094	−1.71	0.088
T1 social skills → T2 extrinsic goals	0.077	0.129	0.60	0.549
**T1 leadership → T2 extrinsic goals**	**−0.258**	0.**129**	**−2.00**	**0.046**

Standardized coefficients are presented. Statistically significant paths are shown in bold. T1, Time 1; T2, Time 2; SE, standard error.

### 3.4 Cross-lagged panel model

A two-wave cross-lagged panel model was estimated using Mplus to examine prospective associations and possible reverse-direction associations between students’ intrinsic and extrinsic goals and the five life-skills factors. The hypothesized model demonstrated acceptable fit to the data, *χ*^2^(970) = 1775.734, CFI = 0.913, TLI = 0.903, RMSEA = 0.049, 90% CI [0.045, 0.053], and SRMR = 0.062. After accounting for prior levels of the corresponding life skills and sex, the unadjusted results indicated positive prospective associations of T1 intrinsic goals with T2 time management (*β* = 0.40, *z* = 3.96, unadjusted *p* < 0.001) and leadership (*β* = 0.46, *z* = 2.22, unadjusted *p* = 0.026). The corresponding paths to teamwork (*β* = 0.52, *z* = 1.67, unadjusted *p* = 0.095), goal setting (*β* = 0.32, *z* = 1.63, unadjusted *p* = 0.103), and social skills (*β* = 0.58, *z* = 1.35, unadjusted *p* = 0.177) were not statistically significant. After applying the Holm correction to the five intrinsic-goal paths, only the association between T1 intrinsic goals and T2 time management remained statistically significant (Holm-adjusted *p* < 0.001). The association with leadership did not survive the correction (Holm-adjusted *p* = 0.104). T1 extrinsic goals were not significantly associated with any of the five life-skills factors either before or after the Holm correction. Regarding the exploratory reverse-direction paths, T1 leadership showed a nominal negative association with T2 extrinsic goals (*β* = −0.26, *z* = −2.00, unadjusted *p* = 0.046). However, this association did not remain statistically significant after correction across the ten reverse-direction tests (Holm-adjusted *p* = 0.460). None of the remaining paths were statistically significant. Unadjusted and Holm-adjusted *p* values for all three inferential families are presented in Supplementary Table S2. Significant autoregressive paths were observed for goal setting (*β* = 0.35, *p* = 0.001), time management (*β* = 0.29, *p* < 0.001), leadership (*β* = 0.24, *p* = 0.018), intrinsic goals (*β* = 0.62, *p* = 0.028), and extrinsic goals (*β* = 0.77, *p* < 0.001). In contrast, the autoregressive paths for teamwork (*β* = 0.21, *p* = 0.271) and social skills (*β* = 0.12, *p* = 0.593) were not statistically significant. Sex was negatively associated with T2 goal setting (*β* = −0.11, *p* = 0.021), leadership (β = −0.11, *p* = 0.026), and extrinsic goals (*β* = −0.11, *p* = 0.024), but was not significantly associated with the remaining T2 variables (all *ps* > 0.05).

### 3.5 Cluster-robust sensitivity analyses

To examine the robustness of the prospective and reverse-direction associations after accounting for the dependency of students within class sections, seven sensitivity analyses were conducted using observed composite scores and cluster-robust standard errors. In the life-skill-specific models, T1 intrinsic goals were positively associated with T2 teamwork (*β* = 0.20, SE = 0.07, *p* = 0.003), goal setting (*β* = 0.15, SE = 0.06, *p* = 0.010), time management (*β* = 0.19, SE = 0.08, *p* = 0.016), and leadership (*β* = 0.16, SE = 0.06, *p* = 0.006) after controlling for the corresponding T1 life skill and sex. The association between T1 intrinsic goals and T2 social skills was positive but not statistically significant (*β* = 0.11, SE = 0.07, *p* = 0.084). T1 extrinsic goals were not significantly associated with any of the five T2 life-skills factors (βs ranged from −0.002 to 0.048, all *ps* > 0.05).

In the goal-content-specific models, T1 teamwork (*β* = 0.12, SE = 0.06, *p* = 0.029) and T1 social skills (*β* = 0.18, SE = 0.06, *p* = 0.003) were positively associated with T2 intrinsic goals after controlling for T1 intrinsic goals and sex. The associations of T1 goal setting, time management, and leadership with T2 intrinsic goals were not statistically significant (βs ranged from −0.11 to 0.03, all *ps* > 0.05). None of the five T1 life skills were significantly associated with T2 extrinsic goals after controlling for T1 extrinsic goals and sex (βs ranged from −0.12 to 0.07, all *ps* > 0.05). Overall, the sensitivity analyses showed only partial convergence with the primary latent CLPM. The prospective association between T1 intrinsic goals and T2 time management was consistently identified across the two specifications, whereas several other prospective and reverse-direction findings differed. Because the models differed in their treatment of measurement error, simultaneous versus outcome-specific estimation, and adjustment for classroom clustering, these discrepancies cannot be attributed exclusively to clustering.

## 4 Discussion

The present study examined the prospective and reverse-direction associations between intrinsic and extrinsic goal contents and five life skills among students enrolled in university physical education courses. After accounting for the prior levels of all constructs and sex, T1 intrinsic goals were positively associated with T2 time management and leadership, but not with teamwork, goal setting, or social skills. After the Holm correction, however, only the prospective association between T1 intrinsic goals and T2 time management remained statistically significant. T1 extrinsic goals were not significantly associated with any of the five life skills at T2. Regarding the reverse-direction associations, T1 leadership showed a nominal negative association with T2 extrinsic goals in the unadjusted results, but this association did not remain statistically significant after the Holm correction. The remaining paths from T1 life skills to T2 intrinsic and extrinsic goals were not statistically significant.

First, the unadjusted results indicated that T1 intrinsic goals were positively associated with T2 time management and leadership. However, only the association with time management remained statistically significant after the Holm correction. Thus, the findings provided limited support for the hypothesis that intrinsic goals would be prospectively associated with all five life skills. These findings were also broadly consistent with previous research linking intrinsic goals to psychological need satisfaction, autonomous behavioral regulation, sustained participation, and adaptive outcomes. In physical activity settings, intrinsic goals have been positively associated with psychological need satisfaction, autonomous behavioral regulation, and participation frequency (Sibley and Bergman, 2018). Longitudinal evidence has further linked intrinsic goal pursuits to subsequent autonomous motivation (Behzadnia et al., 2022; Gunnell et al., 2014). In university physical education, health-management and skill-development goals have also been indirectly associated with subsequent physical activity and physical fitness through autonomous behavioral regulation (Sibley and Bergman, 2016). Beyond physical activity, intrinsic goals have been associated with broader forms of self-regulatory and interpersonal functioning, including mindfulness, prosocial behavior, and pro-environmental behavior (Brown and Kasser, 2005; Fu et al., 2018; Unanue et al., 2016). Collectively, this literature provides a broader theoretical context for the robust prospective association of intrinsic goals with time management and the more tentative unadjusted association with leadership observed in the present study.

According to GCT, intrinsic goals such as skill development, health management, and social affiliation are oriented toward personal growth, health, and meaningful interpersonal relationships (Deci and Ryan, 2000; Kasser and Ryan, 1996). In university physical education, students who pursue skill development and health management may be more likely to plan their activities, allocate limited time, and regulate their efforts toward personally meaningful aims. Participating in this manner may be conceptually linked to time management. Similarly, students who value personal growth and meaningful relationships may be more inclined to take initiative during class activities, support their peers, and assume responsibility for shared tasks. Such experiences may offer one possible explanation for the unadjusted association between intrinsic goals and perceived leadership, although this association did not survive the Holm correction. These findings therefore suggest that, within university physical education, what students seek to attain through their participation may be relevant to time management skills, while the tentative association with leadership requires further replication.

In the primary latent CLPM, T1 intrinsic goals were not significantly associated with T2 teamwork, goal setting, or social skills. Thus, the present study did not identify consistent prospective associations between intrinsic goals and all five life skills. Teamwork and social skills may depend not only on the goal contents pursued by individual students but also on the opportunities for cooperation, peer interaction, and communication provided by the social structure of class activities. For example, when an activity does not sufficiently require cooperation and communication, students may have limited opportunities to experience and demonstrate teamwork and social skills even if they value personal growth or meaningful relationships. Accordingly, associations between intrinsic goals and these interpersonal life skills need to be considered in conjunction with the social experiences afforded by the class environment.

Valuing personal growth or health is also conceptually distinct from possessing the skills required to set specific goals and monitor progress toward them. Consequently, valuing intrinsic goals alone may not necessarily result in goal-setting skills. Students may have more direct opportunities to practice formulating and implementing specific goals when they receive explicit goal-setting instruction, performance feedback, and opportunities for self-monitoring. This distinction highlights the need to differentiate the contents of the goals students pursue from the skills used to plan, implement, and monitor those goals.

Another possibility is that the associations of intrinsic goals with teamwork, goal setting, and social skills involve more complex pathways through psychological processes such as basic psychological need satisfaction or autonomous motivation. Previous research showing that teacher autonomy support was associated with life skills through basic psychological need satisfaction (Cronin et al., 2019, 2022), together with longitudinal evidence linking initial need satisfaction to subsequent life skills (Cronin et al., 2020), provides indirect theoretical support for this possibility. However, teacher autonomy support, as examined in these studies, is conceptually distinct from the goal contents examined in the present study. Furthermore, neither psychological need satisfaction nor autonomous motivation was included in the present two-wave model. The current findings therefore cannot establish whether such indirect pathways were involved. The nonsignificant associations should also not be interpreted as evidence that intrinsic goals are unrelated to teamwork, goal setting, and social skills. Future research should examine these associations while considering the relevant psychological processes and features of the instructional environment.

Second, T1 extrinsic goals were not significantly associated with any of the five life skills at T2. This pattern was broadly consistent with the hypothesis that extrinsic goals would show limited prospective associations with subsequent life skills. According to GCT, extrinsic goals related to body image and social recognition place relatively greater emphasis on external standards such as others’ evaluations, approval, and social comparison than on personal growth or meaningful relationships (Ryan, 2023). In contrast, time management, goal setting, teamwork, social skills, and leadership are closely related to regulating one’s behavior or collaborating effectively with others. This difference in functional emphasis may help explain why no statistically detectable prospective associations were observed between extrinsic goals and subsequent life skills. This interpretation is broadly consistent with previous research showing that appearance- and recognition-related goals are more closely associated with controlled forms of behavioral regulation, such as introjected regulation, than with autonomous regulation (Sebire et al., 2009; Vansteenkiste et al., 2006). More recent research has also linked the pursuit of extrinsic goals to controlled motivation (Behzadnia et al., 2022; Jeno et al., 2018) and basic psychological need frustration (Bradshaw et al., 2021; Nishimura et al., 2021). Longitudinal evidence has also shown that attaining extrinsic aspirations was not associated with wellbeing and was instead related to greater ill-being (Niemiec et al., 2009). This literature suggests that students who prioritize external evaluation and approval may participate in physical activities to improve their appearance or gain recognition from others, but such goals may not necessarily be directly connected to how they experience and use time management, cooperation, communication, and leadership.

Importantly, the absence of significant associations between extrinsic goals and life skills does not indicate that extrinsic goals were negatively associated with life skills. Intrinsic and extrinsic goals represent conceptually distinct goal contents rather than opposite ends of a single continuum (McLachlan and Hagger, 2011), and the meaning and outcomes of extrinsic goals may vary according to the reasons underlying their pursuit and the instructional context in which they are presented (Sheldon et al., 2004). In school physical education, conditions that emphasized extrinsic goals did not consistently undermine students’ motivation or lesson experiences; in some conditions, students instead reported greater perceived lesson value and stronger intentions for future participation (Gillison et al., 2013). Because the effects of the instructional conditions in that study were limited and assessed immediately after a single lesson, these findings should not be interpreted as evidence of sustained benefits from extrinsic goals. Evidence that extrinsic goals provide little additional motivational benefit and may carry modest costs when combined with strong intrinsic goal endorsement further supports the view that intrinsic and extrinsic goals do not function as simple opposites (Lindwall et al., 2016). Accordingly, extrinsic goals may not be inherently detrimental; their meaning and outcomes may depend on the motivational and instructional contexts in which they are pursued. The present findings similarly indicate not that extrinsic goals were associated with life skills in the direction opposite to intrinsic goals, but that no significant prospective associations were identified between extrinsic goals and any of the five life skills over one academic semester. Future research should therefore consider not only goal contents but also the reasons underlying their pursuit and the instructional contexts in which they are emphasized.

Among the exploratory reverse-direction paths, T1 leadership showed a nominal negative association with T2 extrinsic goals in the unadjusted results. However, this association did not remain statistically significant after the Holm correction across the ten reverse-direction tests. Accordingly, the finding does not provide robust evidence of a reverse-direction association and should be regarded as preliminary. None of the remaining paths from life skills to subsequent intrinsic or extrinsic goals were statistically significant. Nevertheless, the nonsignificant findings do not establish the absence of reverse-direction associations. Future studies incorporating additional measurement waves should therefore examine whether the leadership finding can be replicated and whether other reverse-direction associations emerge over longer periods.

The cluster-robust sensitivity analyses provided partial support for the findings of the primary latent CLPM while also revealing some differences across model specifications. The prospective association between intrinsic goals and time management was consistently identified across both approaches and remained statistically significant after the Holm correction in the primary latent CLPM. At the unadjusted level, both approaches also identified an association between intrinsic goals and leadership. However, this association did not survive the Holm correction in the latent CLPM. The cluster-robust analyses additionally identified positive associations of intrinsic goals with teamwork and goal setting, which were not statistically significant in the latent CLPM. Differences were also observed in the reverse-direction paths. Whereas the latent CLPM showed a nominal unadjusted negative association between leadership and subsequent extrinsic goals, this association did not survive the Holm correction and was not identified in the cluster-robust analyses. Conversely, teamwork and social skills were positively associated with subsequent intrinsic goals only in the cluster-robust analyses. These differences may reflect the distinct characteristics of the two analytical specifications. The latent CLPM accounted for measurement error and estimated all autoregressive, prospective, and reverse-direction paths simultaneously, whereas the sensitivity analyses used observed composite scores and estimated separate, more parsimonious outcome-specific models while adjusting standard errors for classroom clustering. Thus, the association between intrinsic goals and time management showed the clearest convergence across the two approaches, whereas the remaining path-specific findings were model-dependent and require replication using a larger number of class clusters and multilevel latent-variable models. Despite these model-specific differences, the present study contributes to the literature by connecting goal contents and life skills within a single prospective framework. Previous studies have largely examined goal contents in relation to motivational and wellbeing outcomes (Behzadnia and Deci, 2026; McLachlan and Hagger, 2011; Sibley and Bergman, 2016) or life skills in physical activity settings (Cronin et al., 2018, 2019; Holt et al., 2017) as separate lines of inquiry. By connecting these two areas, the present study suggests that goal contents may offer a perspective on life skills that is conceptually distinct from that provided by motivational regulation. However, because motivational regulation and psychological need satisfaction were not assessed, the independent contribution of goal contents relative to these processes could not be established.

## 5 Practical implications

From a practical standpoint, the findings point to the potential value of supporting intrinsic goal contents in university physical education, particularly in relation to students’ perceived time management. The tentative association with leadership requires further investigation. Educators and coaches may support students in identifying personally meaningful goals related to skill development, health management, and social affiliation, while emphasizing mastery, effort, and self-improvement over appearance or social recognition. This does not mean that instructors should prescribe intrinsic goals or discourage students from pursuing appearance- or recognition-related goals. Rather, they may encourage students to consider how participation can also contribute to personally meaningful aims such as improving their health, developing their skills, and building meaningful relationships. Supporting intrinsic goal contents requires more than simply telling students that growth, health, and relationships are important. Students need opportunities to connect course participation with intrinsic goals that they personally value. As broader SDT-informed guidance rather than practices directly tested in the present study, a recently developed classification of SDT-based teaching behaviors recommends helping students connect learning activities with intrinsic life goals, providing meaningful choices and rationales, and discussing how class activities relate to values that students consider important. It also recommends supporting competence through optimally challenging tasks, self-referenced goals, specific improvement-oriented feedback, clear action plans, and opportunities for self-monitoring, while supporting relatedness by acknowledging students’ perspectives and promoting cooperation (Ahmadi et al., 2023; Vansteenkiste et al., 2004).

Applied to university physical education, instructors could invite students to identify whether they wish to improve their health, develop their skills, or build meaningful relationships through course participation. Students could then translate these intrinsic goals into personally relevant participation plans, monitor their effort and progress, adjust their plans in response to difficulties, and assume responsibility for group tasks or rotating leadership roles. At the same time, personally valuing intrinsic goals alone may not be sufficient to support every life skill. Instructors may also need to provide explicit and repeated opportunities to practice particular skills through structured goal-setting tasks, cooperative activities, peer communication, leadership assignments, and guided reflection. Instructors may encourage students to reflect on how the planning, communication, cooperation, and leadership practiced in class could be applied in academic, interpersonal, and future occupational contexts. However, the present findings do not establish that experimentally promoting intrinsic goal contents causes improvements in life skills. Intervention research is therefore needed to determine whether instructional strategies designed to foster intrinsic goal contents lead to subsequent improvements in students’ perceived life skills. Because the present study did not assess students’ actual use of life skills or their transfer beyond physical education, whether these strategies facilitate the application of life skills across contexts should also be examined directly in future research.

## 6 Limitations and future research

Several limitations warrant consideration when interpreting the present findings. First, all constructs were assessed exclusively through self-report measures. Although self-report instruments are widely used in motivational and developmental research, reliance on a single method increases susceptibility to common method variance and shared response tendencies. Moreover, the life-skills measure assessed students’ perceptions of their skills rather than objectively demonstrated competencies, actual skill use, or transfer beyond physical education. The latent-factor correlations and HTMT results indicated substantial shared variance among some life-skills domains, particularly between social skills and leadership at T2, where the HTMT value exceeded the conservative 0.85 criterion. This overlap may have increased parameter uncertainty and reduced the precision of the unique cross-lagged estimates, potentially contributing to the relatively large but nonsignificant coefficients from intrinsic goals to teamwork and social skills. Future research would benefit from incorporating multimethod assessments, including behavioral indicators, peer or instructor evaluations, and experience-sampling methodologies, to enhance construct validity and reduce potential response bias. Assessments across academic, interpersonal, and occupational contexts would also help determine whether perceived life skills are enacted across settings.

Second, the study employed a two-wave cross-lagged panel design spanning a single academic semester. Although this design permits examination of prospective associations, it does not establish causality or disentangle stable between-person differences from within-person fluctuations over time (Hamaker et al., 2015). Consequently, neither the significant nor the nonsignificant paths should be interpreted as evidence of causal effects or the presence or absence of within-person processes. In addition, the complete latent CLPM contained too many freely estimated parameters relative to the 26 class clusters to obtain reliable cluster-robust standard errors. Although outcome-specific sensitivity analyses adjusted for class-level dependency, they were not equivalent to the complete latent reciprocal model. Future studies should incorporate three or more waves and apply advanced longitudinal approaches, such as random-intercept cross-lagged panel models or multilevel latent-variable models, while including a larger number of class sections. Experimental and intervention designs are also needed before concluding that promoting intrinsic goal contents causes improvements in perceived life skills.

Third, although the retention rate was relatively high (88.3%), linkable baseline data for participants who were not retained at T2 were unavailable, preventing comparisons between retained participants and those lost to follow-up. Systematic attrition therefore cannot be ruled out. Moreover, the sample was drawn exclusively from university students participating in physical education courses within a specific educational and cultural context. Although this context is developmentally meaningful during emerging adulthood, the generalizability of the findings to other universities, cultural settings, and physical activity contexts remains uncertain. Contextual characteristics such as autonomy support, motivational climate, competitive intensity, and instructional structure may condition the observed associations but were not directly examined in the present study. Because the 12 activity types were unevenly distributed across only 26 class sections, activity-type effects could not be reliably distinguished from class-section and instructor characteristics. Replication in different universities and cultural settings and across other physical activity contexts, including recreational sport, community-based exercise, and elite athletics, is therefore needed to establish the boundary conditions of the observed associations.

Fourth, intrinsic and extrinsic goals were modeled as broader goal-content factors, which may have obscured distinct associations of skill development, health management, social affiliation, body image, and social recognition with particular life skills. In addition, the present study did not directly examine the psychological mechanisms through which goal contents were prospectively associated with perceived life skills. Although GCT suggests that intrinsic goals may be associated with adaptive functioning through processes such as psychological need satisfaction, the present design did not include potential mediating variables. Psychological need satisfaction, psychological need frustration, and behavioral regulation were not assessed. Therefore, the proposed roles of need satisfaction and autonomous motivation in explaining the prospective findings involving intrinsic goals, as well as the possible roles of need frustration and controlled motivation in understanding the nonsignificant associations involving extrinsic goals, should be regarded as theoretically plausible explanations rather than tested mechanisms. Consequently, the mechanisms underlying the observed prospective associations remain unclear. Future research should examine individual goal contents and these potential mechanisms across at least three measurement occasions or within experimental or intervention designs.

## 7 Conclusion

The present study examined the prospective associations between goal contents and life skills, as well as possible reverse-direction associations, among students enrolled in university physical education courses. The clearest and most consistent finding was the positive prospective association between intrinsic goals and subsequent time management. No statistically detectable prospective associations were observed for extrinsic goals, and the exploratory results did not provide robust evidence of reverse-direction associations.

Taken together, the findings extend the application of GCT to university physical education by showing that the pattern of prospective associations between goal contents and life skills was not uniform across the five dimensions. The absence of significant negative associations between extrinsic goals and subsequent life skills also does not support interpreting extrinsic goals as either the simple opposite of intrinsic goals or inherently detrimental to life skills. More broadly, the study highlights the relevance of considering what students seek to attain through participation when examining their experiences of life skills in university physical education. However, the findings do not demonstrate that promoting intrinsic goal contents causes improvements in life skills or that these skills are enacted and transferred beyond physical education. Further research using additional measurement waves and experimental or intervention designs is needed to examine these possibilities.

## Data Availability

The raw data supporting the conclusions of this article will be made available by the authors, without undue reservation.
