# Supplementary material for: What students pursue matters: longitudinal associations between goal contents and life skills in university physical education
Source: Front Psychol. 2026 Sep 25;17:1964768. doi: 10.3389/fpsyg.2026.1964768 (PMC13649727; doi:10.3389/fpsyg.2026.1964768)
Supplement: Supplementary file 1 [file Supplementary_file_1.docx]

**Supplementary Table S1**
Intraclass correlation coefficients and design effects for the principal study variables

| **Variable** | **T1 ICC** | **T1 design effect** | **T2 ICC** | **T2 design effect** |
| --- | --- | --- | --- | --- |
| Intrinsic goals | .019 | 1.24 | .006 | 1.08 |
| Extrinsic goals | .000 | 1.00 | .057 | 1.70 |
| Teamwork | .052 | 1.64 | .014 | 1.17 |
| Goal setting | .050 | 1.62 | .000 | 1.00 |
| Time management | .116 | 2.43 | .000 | 1.00 |
| Social skills | .060 | 1.74 | .027 | 1.33 |
| Leadership | .092 | 2.13 | .034 | 1.42 |

**Note.** T1 = Time 1; T2 = Time 2; ICC = intraclass correlation coefficient. ICCs were estimated from unconditional random-intercept models with students nested within 26 class sections. Design effects were calculated as 1 + (m̄ − 1) × ICC, where m̄ = 13.35 was the average class size.

**Supplementary Table S2**

Unadjusted and Holm-adjusted p values for the prospective and reverse-direction paths in the latent cross-lagged panel model.

| **Inferential family** | **Path** | **Unadjusted p** | **Holm-adjusted p** |
| --- | --- | --- | --- |
| Intrinsic-goal paths | IG_T1 → TM_T2 | < .001 | < .001 |
|  | IG_T1 → LS_T2 | .026 | .104 |
|  | IG_T1 → TW_T2 | .095 | .285 |
|  | IG_T1 → GS_T2 | .103 | .285 |
|  | IG_T1 → SS_T2 | .177 | .285 |
| Extrinsic-goal paths | EG_T1 → TW_T2 | .403 | 1.000 |
|  | EG_T1 → SS_T2 | .556 | 1.000 |
|  | EG_T1 → TM_T2 | .559 | 1.000 |
|  | EG_T1 → LS_T2 | .619 | 1.000 |
|  | EG_T1 → GS_T2 | .624 | 1.000 |
| Reverse-direction paths | LS_T1 → EG_T2 | .046 | .460 |
|  | LS_T1 → IG_T2 | .064 | .576 |
|  | TM_T1 → EG_T2 | .088 | .704 |
|  | GS_T1 → EG_T2 | .225 | 1.000 |
|  | SS_T1 → IG_T2 | .235 | 1.000 |
|  | TW_T1 → EG_T2 | .301 | 1.000 |
|  | TM_T1 → IG_T2 | .536 | 1.000 |
|  | SS_T1 → EG_T2 | .549 | 1.000 |
|  | TW_T1 → IG_T2 | .695 | 1.000 |
|  | GS_T1 → IG_T2 | .713 | 1.000 |

**Note.** IG = intrinsic goals; EG = extrinsic goals; TW = teamwork; GS = goal setting; TM = time management; SS = social skills; LS = leadership; T1 = Time 1; T2 = Time 2. Holm corrections were applied separately to three theoretically defined inferential families: (a) the five prospective paths from T1 intrinsic goals to T2 life skills, (b) the five prospective paths from T1 extrinsic goals to T2 life skills, and (c) the ten exploratory reverse-direction paths from T1 life skills to T2 intrinsic and extrinsic goals. Holm-adjusted p values greater than 1 were reported as 1.000. After correction, only the path from T1 intrinsic goals to T2 time management remained statistically significant.

**Supplementary Table S3**

Latent-factor correlations and heterotrait–monotrait ratios among the five life-skills factors at T1 and T2.

Panel A. Time 1

| Factor | TW | GS | TM | SS | LS |
| --- | --- | --- | --- | --- | --- |
| TW | 1.000 | .730 | .586 | .713 | .681 |
| GS | .712 | 1.000 | .773 | .721 | .703 |
| TM | .573 | .745 | 1.000 | .598 | .645 |
| SS | .695 | .685 | .554 | 1.000 | .840 |
| LS | .664 | .695 | .640 | .813 | 1.000 |

Panel B. Time 2

| Factor | TW | GS | TM | SS | LS |
| --- | --- | --- | --- | --- | --- |
| TW | 1.000 | .779 | .618 | .821 | .699 |
| GS | .757 | 1.000 | .780 | .765 | .786 |
| TM | .587 | .761 | 1.000 | .679 | .758 |
| SS | .809 | .733 | .626 | 1.000 | .883 |
| LS | .685 | .782 | .728 | .852 | 1.000 |

**Note.** Latent-factor correlations are presented below the diagonal, and heterotrait–monotrait ratios (HTMT) are presented above the diagonal. TW = teamwork; GS = goal setting; TM = time management; SS = social skills; LS = leadership; T1 = Time 1; T2 = Time 2. All latent-factor correlations were statistically significant at *p* < .001.
